# Effectiveness of implementation strategies for increasing clinicians’ use of five validated imaging decision rules for musculoskeletal injuries: a systematic review

**DOI:** 10.1186/s12873-024-00996-x

**Published:** 2024-05-17

**Authors:** Priti Kharel, Joshua R. Zadro, Grace Wong, Kittirut Rojanabenjawong, Adrian Traeger, James Linklater, Christopher G. Maher

**Affiliations:** 1 The University of Sydney, Sydney Musculoskeletal Health, Sydney, NSW Australia PO Box M179, Priti Kharel - Level 10 North, King George V Building, Royal Prince Alfred Hospital, Missenden Road, 2050; 2 Sydney School of Public Health, The University of Sydney, Sydney, NSW Australia; 3 Castlereagh Imaging, Sydney, NSW Australia

**Keywords:** Canadian C-spine Rule, Canadian CT Head Rule, NEXUS guidelines, Ottawa Ankle Rules and Ottawa knee Rule

## Abstract

**Background:**

Strategies to enhance clinicians’ adherence to validated imaging decision rules and increase the appropriateness of imaging remain unclear.

**Objective:**

To evaluate the effectiveness of various implementation strategies for increasing clinicians’ use of five validated imaging decision rules (Ottawa Ankle Rules, Ottawa Knee Rule, Canadian C-Spine Rule, National Emergency X-Radiography Utilization Study and Canadian Computed Tomography Head Rule).

**Design:**

Systematic review.

**Methods:**

The inclusion criteria were experimental, quasi-experimental study designs comprising randomised controlled trials (RCTs), non-randomised controlled trials, and single-arm trials (i.e. prospective observational studies) of implementation interventions in any care setting. The search encompassed electronic databases up to March 11, 2024, including MEDLINE (via Ovid), CINAHL (via EBSCO), EMBASE (via Ovid), Cochrane CENTRAL, Web of Science, and Scopus. Two reviewers assessed the risk of bias of studies independently using the Cochrane Effective Practice and Organization of Care Group (EPOC) risk of bias tool. The primary outcome was clinicians’ use of decision rules. Secondary outcomes included imaging use (indicated, non-indicated and overall) and knowledge of the rules.

**Results:**

We included 22 studies (5-RCTs, 1-non-RCT and 16-single-arm trials), conducted in emergency care settings in six countries (USA, Canada, UK, Australia, Ireland and France). One RCT suggested that reminders may be effective at increasing clinicians’ use of Ottawa Ankle Rules but may also increase the use of ankle radiography. Two RCTs that combined multiple intervention strategies showed mixed results for ankle imaging and head CT use. One combining educational meetings and materials on Ottawa Ankle Rules reduced ankle injury imaging among ED physicians, while another, with similar efforts plus clinical practice guidelines and reminders for the Canadian CT Head Rule, increased CT imaging for head injuries. For knowledge, one RCT suggested that distributing guidelines had a limited short-term impact but improved clinicians’ long-term knowledge of the Ottawa Ankle Rules.

**Conclusion:**

Interventions such as pop-up reminders, educational meetings, and posters may improve adherence to the Ottawa Ankle Rules, Ottawa Knee Rule, and Canadian CT Head Rule. Reminders may reduce non-indicated imaging for knee and ankle injuries. The uncertain quality of evidence indicates the need for well-conducted RCTs to establish effectiveness of implementation strategies.

**Supplementary Information:**

The online version contains supplementary material available at 10.1186/s12873-024-00996-x.

## Introduction

Imaging decision rules are tools that can increase the appropriateness of imaging requests [1] by guiding clinicians on when imaging studies, such as X-rays or CT scans, are indicated (e.g. for suspected serious conditions or injuries) and when they are unnecessary (e.g. for less concerning conditions or injuries). Several validated imaging decision rules can help clinicians differentiate patients at high or low risk of having a serious pathology (e.g., fracture) following a musculoskeletal injury [2]. These include the Canadian CT Head Rule, Canadian C-spine Rule, National Emergency X-Radiography Utilization Study (NEXUS) guideline, Ottawa Ankle Rules and Ottawa Knee Rule [3–6]. The Canadian CT Head Rule can help clinicians identify patients at low risk of brain injury and who do not require a CT scan (99–100% sensitivity) [2]. The Canadian C-spine Rule (99–100% sensitivity) [3] and the NEXUS guideline (83–100% sensitivity) [5] assess potential cervical spine injuries using criteria including cervical spine tenderness, level of alertness, neurological deficits, evidence of intoxication, painful distracting injuries, patient’s age, mechanism of injury, neck mobility and numbness in arms or legs. The Ottawa Ankle Rules (99.4% sensitivity) [3] and Ottawa Knee Rule (98.5% sensitivity) [4] determine the necessity for ankle and knee X-rays, respectively, through criteria such as weight-bearing ability and tenderness. Use of these rules can help ensure clinicians do not miss serious injuries while avoiding unnecessary or non-indicated imaging [7–9].

Several studies have investigated the effectiveness of strategies to increase clinicians’ use of validated imaging decision rules. However, results are conflicting possibly due to variations in the target population. For example, one single-arm trial found that educational meetings, reminders, and audit and feedback increased emergency department (ED) clinicians’ (Triage nurses, Emergency nurse practitioners, and Medical staff) use of the Ottawa Ankle Rules [1] and reduced use of ankle X-rays. However, another study found educational meetings and materials about the Ottawa Ankle Rules increased clinicians’ (physician assistants, residents, and attending physicians) use of the rules but did not reduce use of imaging [10].

Due to differing results in the literature and no previous review on this topic, a systematic review is needed to identify strategies that can increase clinicians’ use of validated imaging decision rules for musculoskeletal injuries and improve the appropriateness of imaging. The primary aim of this systematic review was to evaluate the effect of various implementation strategies on clinicians’ use of five validated imaging decision rules (Ottawa Ankle Rules, Ottawa Knee Rule, Canadian C-Spine Rule, National Emergency X-Radiography Utilization Study and Canadian Computed Tomography Head Rule). The secondary aims were to evaluate the effect of implementation strategies on imaging use (indicated, non-indicated and overall) and clinician knowledge.

## Methods

We conducted the systematic review in accordance with the “Preferred reporting items for systematic reviews and meta-analyses” (PRISMA) statement [11], and the protocol was registered prospectively on PROSPERO (CRD42020150131). We also followed guidelines from the Effective Practice and Organization of Care (EPOC) group for the conduct of a systematic review of implementation strategies [12].

### Search strategy

The following electronic databases were searched from the earliest record until March 11, 2024: MEDLINE (via Ovid), CINAHL (via EBSCO), EMBASE (via Ovid), Cochrane Central Register of Controlled Trials (CENTRAL), Web of Science and Scopus. We consulted a librarian to develop the search strategy and used a combination of keywords related to the five decision rules (Ottawa Ankle Rules, Ottawa Knee Rule, Canadian C-Spine Rule, National Emergency X-Radiography Utilization Study and Canadian Computed Tomography Head Rule) (Supplementary File 1). We also performed citation tracking and hand-searching the reference lists of included studies to identify studies missed by the primary electronic database search.

 Two reviewers (PK and JRZ) independently familiarised themselves with the inclusion/exclusion criteria and performed the selection of studies by subsequently screening the title, abstract, and full text of studies retrieved through our electronic database searches. Discrepancies were resolved through discussion or consultation with a third reviewer (CGM).

### Inclusion and exclusion criteria

#### Study design

We included experimental and quasi-experimental study designs (e.g., randomised controlled trials, non-randomised controlled trials) and single-arm trials (i.e. prospective observational studies) of implementation interventions in any care setting. Retrospective and cross-sectional observational studies, case series and case studies were excluded. There were no language or geographic restrictions.

#### Participants

Participants were healthcare professionals involved in the management of people with musculoskeletal injuries in any care setting (e.g., general practitioners, ED physicians, physiotherapists, ED nurses).

#### Interventions and comparators

We included studies that investigated the effectiveness of any intervention that aimed to increase clinicians’ use of the Canadian CT Head Rule, Canadian C-spine Rule, NEXUS guidelines, Ottawa Ankle Rules or Ottawa Knee Rule. The EPOC Intervention Taxonomy was used to classify the types of implementation strategies used in each study [13]. Examples of implementation strategies included the distribution of clinical practice guidelines, reminders, interactive educational meetings, audit and feedback, distribution of educational materials, patient-mediated interventions (e.g. any intervention aimed at changing the performance of healthcare professionals through interactions with patients, or information provided by or to patients) [13], and monitoring the performance of the delivery of healthcare. Both single and multi-component interventions were included. No restriction was placed on the comparison intervention (e.g., another implementation strategy, usual care, no intervention).

#### Outcomes

The primary outcome was clinicians’ use of decision rules. Study investigators could have assessed use of the rules either by clinician self-report (via clinician surveys) or by audits of the clinical notes (documented use of the rules (yes/no) or documented clinical features suggesting use of the rules (yes/no)). Secondary outcomes included use of imaging (e.g., X-ray, CT) as assessed by audits of clinical notes and treatment recording forms, and knowledge of the rules as assessed by surveys. Use of imaging was categorised as indicated, non-indicated and overall. Documentation of clinical features consistent with imaging decision rules was used to determine whether imaging was indicated or not. Table 1 explains the outcomes in more detail.

**Table 1:** Definitions of variables for data extraction

Variables	Definitions
Use of rules	These data were captured in three different ways. (1) Surveys of clinicians provided data on the number of clinicians who reported using the rules (e.g., Do you currently use this rule? Yes/No). (2) Audits of clinical notes were conducted to obtain data on the number of patients whose notes indicated the use of an imaging decision rule to guide imaging decisions. (3) Audits of clinical notes were conducted to identify clinical features mentioned in the notes that would suggest the use of an imaging decision rule to guide imaging decisions.
Use of imaging (indicated, non-indicated and overall)	These data were captured through audits of clinical notes or treatment recording forms. Indicated imaging refers to the number of imaging tests performed in alignment with specific decision rules, indicating that an imaging study is necessary based on the patient’s clinical presentation and characteristics. Non-indicated imaging refers to imaging tests that are conducted even when the decision rules suggest that an imaging study is not required for the particular patient.
Knowledge	In our review, knowledge of the rules was defined as understanding the assessment criteria (or items) of each rule. This is distinct from awareness which is related to being aware of the existence of the rules [14]. Knowledge was captured through questions about knowledge of the rules and their components (e.g., assessing participants’ knowledge of the Ottawa Ankle Rules using questionnaires and a scoring system based on specific criteria for ankle and foot components of the rules).

### Data extraction and quality assessment

Two reviewers (GW and KR) independently used a standardised form, developed collaboratively by three of the authors: PK, JZ, and CM (Supplementary Table 1) to extract data on country, study design and setting, sample size, participant characteristics, implementation strategy (and comparison), and outcomes. Disagreements were resolved by discussion and re-checking the study report. Four authors were contacted to obtain full text or additional data, but they did not respond.

Two reviewers (GW and KR) independently assessed the risk of bias of included studies using the Cochrane EPOC risk of bias tool [12]. This tool was specifically developed to assess the risk of bias in studies investigating strategies to change the practice of healthcare providers. The reviewers judged a study to be at ‘low-risk’, ‘high-risk’ or ‘unclear risk’ of bias for the following domains: random sequence generation, allocation concealment, baseline outcome measurements similar, baseline characteristics similar, incomplete outcome data, knowledge of the allocated interventions adequately prevented during the study, protection against contamination, selective outcome reporting, and other risk of bias. Judgments were based on how the identified bias would influence the results of the study. Disagreements in ratings were resolved by a third reviewer (PK).

### Data analysis

Due to the heterogeneity of interventions and outcome measures, findings were not pooled across studies. Instead, a narrative synthesis of published results was performed. We did not apply the Grading of Recommendations Assessment, Development, and Evaluation (GRADE) [15] approach to assess the overall quality of evidence and strength of recommendation as we could not provide a summary measure for any intervention effect.

### Patient and public involvement

We did not involve patients and members of the public in the design of this study.

## Results

### Study characteristics

**Fig. 1:**
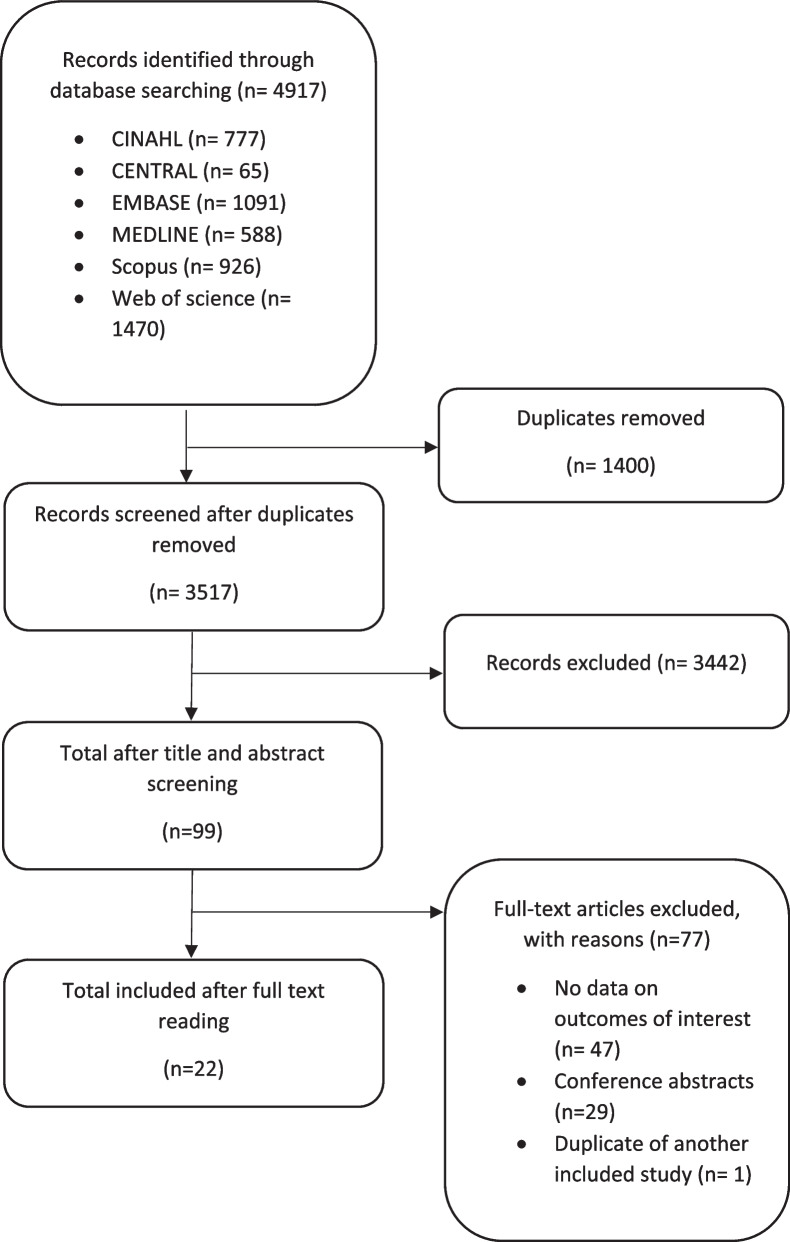
Preferred Reporting Items for Systematic Reviews and Meta-Analyses (PRISMA) flow diagram

After removing duplicates and screening 3517 titles and abstracts and 99 full-text reports, 22 studies were included (Fig. 1). This included five RCTs, one non-randomised controlled trial, and 16 single-arm trials. Of the included studies, 10 focus on the Ottawa Ankle Rules, five on the Canadian CT Head Rule, three on the Ottawa Knee Rule, three on the NEXUS guidelines and two on the Canadian C-spine Rule. Ten studies reported data on use of rules, 14 on overall imaging use, six on indicated or non-indicated imaging and two on knowledge of the rules. The studies provided data from the following countries: 10 from the USA, six from Canada, two from the UK, two from Australia, one from France and one from Ireland. The study settings included 17 community/tertiary/teaching hospital EDs, three acute care centres and two major trauma centres. The review involved 1,271 clinicians and 35,010 patients. The included types of clinicians were ED physicians in 13 studies, junior doctors in 10 studies, ED nurse practitioners in 6 studies, physician assistants in 4 studies, ED nurses in 4 studies, physiotherapists and trauma team leaders in 1 study each (Table 2). Some studies included multiple clinician types. Detailed characteristics of the included studies is shown in Supplementary Tables 2 and a summary of main findings are shown in Table 3.

**Table 2:** Study characteristics summary

	Number of studies
**Study types**	
Single-arm trials	16
RCT	5
Non-RCT	1
**Decision rules**	
Ottawa Ankle Rules	10
Canadian CT Head Rule	5
Ottawa Knee Rule	3
NEXUS guidelines^a^	3
Canadian C-Spine rules^a^	2
**Outcomes of interest**	
Overall imaging use	14
Use of rules	10
Indicated or non-indicated imaging	6
Knowledge of the rules	2
**Countries**	
USA	10
Canada	6
UK	2
Australia	2
France	1
Ireland	1
**Hospital settings**	
Community/tertiary/teaching hospital EDs,	17
Acute care centres	3
Major trauma centres, and	2
**Participants**	
Clinicians	1271^b^
Patients	35,010^b^
**Clinician types**	
ED physicians	13
Junior doctors	10
ED nurse practitioners	6
Physician assistants	4
ED nurses	4
Trauma team leaders	1

^a^ one study reported data on both the Canadian C-spine Rules and NEXUS guidelines

^b^ total sample size

**Table 3:**
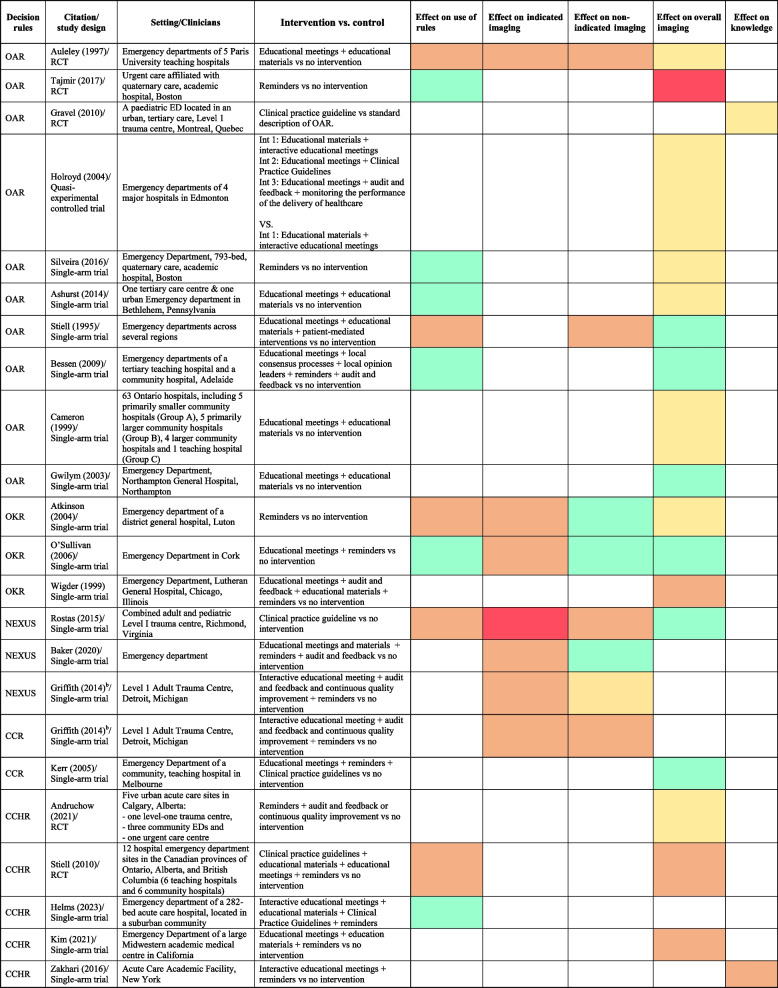
Summary of main findings

* Green  - desired effect (increased use/decreased non-indicated use/increased indicated imaging/decreased overall imaging), Red - undesired effect, Yellow - no effect, Orange – unknown effect due to no *p*-value reported/incomplete data on control or intervention, blank – not applicable

### Risk of bias

Supplementary Table 3 presents the risk of bias scores for each study according to the Cochrane EPOC tool. Three studies were at low risk of bias for all but one domain (random sequence generation [16], protection against contamination [17] and selective outcome reporting [18]). The key findings from our risk of bias assessment are that 16 studies were at high risk of bias for random sequence generation and allocation concealment, 15 for ‘knowledge of allocated interventions,’ and 13 for protection against contamination. On the other hand, 18 studies were at low risk of bias for selective outcome reporting, 13 for protection against contamination, 12 for ‘other bias,’ and 8 for ‘baseline characteristics similar.’ The biases identified could potentially affect the validity and reliability of the study results. The table also shows high variability across studies and highlights areas that require caution when interpreting the findings.

### Use of rules (primary outcome)

#### Ottawa Ankle Rules

One RCT found pop-up reminders in electronic medical records increased medical doctors’ and physician assistants’ use of the Ottawa Ankle Rules compared to no intervention (93% vs. 62%, *p* = 0.02) [19]. Another RCT compared the effectiveness of educational materials related to Ottawa Ankle Rules combined with educational meetings against no intervention and the use of rules in the intervention group was reported as 93% [16]. The study, however, was missing data on the control group’s use of rules (Table 4).

**Table 4:** Summary of results from included studies

Decision rule	Study design	Study	Study setting	Sample size	Intervention vs. control	Results
**Use of rules - assessed by documentation of clinical features consistent with rules**						
OAR	RCT	Tajmir (2017)^a^	Urgent care affiliated with quaternary care, academic hospital, Boston	613 patients (258 in pre-intervention period and 374 in post-intervention period)	Reminders vs. no intervention	**Control vs. Intervention** **Ankle Rules** 231/374 (62%) vs. 239/258 (93%), *p* = 0.02 **Foot rules** 238/374 (64%) vs. 209/258 (81%), *p* < 0.01
		Auleley (1997)^b^	Emergency departments of 5 Paris University teaching hospitals	4129 patients (1992 in the intervention and 2137 in the control group)	Educational meetings + educational materials vs. no intervention	**Intervention phase (no data for control )** Intervention group (no n reported) 93%
	Single-arm trial	Silveira (2016)	Emergency Department, 793-bed, quaternary care, academic hospital, Boston	460 ED visits for 45 patients (205 in pre-intervention period and 255 in post-intervention period)	Reminders vs. no intervention	**Pre vs. Post-intervention** 229/410 (56%) vs. 488/510 (96%), *p* < 0.001
		Ashurst (2014)	One tertiary care centre & one urban Emergency department in Bethlehem, Pennsylvania	60 patients (30 in pre-intervention period and 30 in post-intervention period)	Educational meetings + educational materials vs. no intervention	**At triage (pre- vs. post-intervention)** 1/30 (3%) vs. 2/30 (7%), *p* < 0.001 **After triage (pre- vs. post-intervention)** 6/30 (20%) vs. 25/30 (83%), *p* < 0.001
		Stiell (1995)	Emergency departments across several regions	1,276,288 patients (30 in pre-intervention period and 6489 in post-intervention period)	Educational meetings + educational materials + patient-mediated interventions vs. no intervention	**Intervention phase (no pre-intervention data)** **Post-intervention** - Ankle *n* = 4768/5003 (95%) - Foot *n* = 4753/5003 (95%)
		Bessen (2009)	Emergency departments of a tertiary teaching hospital and a community hospital, Adelaide	1561 patients (459 in pre-intervention period and 1102 in post-intervention period)	Educational meetings + local consensus processes + local opinion leaders + reminders + audit and feedback vs. no intervention	**Request forms (pre- vs. post-intervention)** - Tertiary hospital 84/205 (41%) vs. 588/707 (83%), *p* < 0.001 - Community hospital 76/223 (34%) vs. 147/225 (65%), *p* < 0.001 **Case notes (pre- vs. post-intervention)** - Tertiary hospital 123/214 (58%) vs. 767/810 (95%), *p* < 0.001 - Community hospital 216/244 (52%) vs. 231/286 (81%), *p* < 0.001
OKR	Single-arm trial	Atkinson (2004)	Emergency department of a district general hospital, Luton	130 patients (58 in pre-intervention period and 72 in post-intervention period)	Reminders vs. no intervention	**Pre vs. Post-intervention** 44/58 (76%) vs. 67/72 (93%) (*p*-value not provided)
		O’Sullivan (2006)	Emergency Department in Cork	79 patients (43 in pre-intervention period and 36 in post-intervention period)	Educational meetings + reminders vs. no intervention	**Pre vs. Post-intervention** 10/29 (35.5%) vs. 14/23 (61%), *p* = 0.05
NEXUS	Single-arm trial	Rostas (2015)	Combined adult and pediatric Level I trauma centre, Richmond, Virginia	233 patients (128 in pre-intervention period and 105 in post-intervention period)	Clinical practice guideline vs. no intervention	**Pre vs. Post-intervention** 46/54 (85%) vs. 24/30 (80%) (*p*-value not provided)
CCHR	RCT	Stiell (2010)^b^	12 hospital emergency department sites in the Canadian provinces of Ontario, Alberta, and British Columbia (6 teaching hospitals and 6 community hospitals)	4531 patients (2580 in intervention group and 1951 in control group)	Clinical practice guidelines + educational materials + educational meetings + reminders vs. no intervention	**Post-intervention (no data for control)** Intervention group 909/1166 (78%)
	Single-arm trial	Helms (2023)	Emergency department of a 282-bed acute care hospital, located in a suburban community	600 medical records	Interactive educational meetings + educational materials + Clinical Practice Guidelines + reminders	**Pre vs. Post-intervention** 171/264 (64.6%) vs. 249/336 (74.3%), *p* = 0.01
**Indicated and non-indicated imaging**						
OAR	RCT	Auleley (1997)	Emergency departments of 5 Paris University teaching hospitals	4129 patients (1992 in the intervention and 2137 in the control group)	Educational meetings + educational materials vs. no intervention	**Intervention group**^**c**^ **Indicated radiographs (n not reported)** 98.5% **Non-indicated radiographs (n not reported)** 20.5%
	Single-arm trial	Stiell (1995)	Emergency departments across several regions	1,276,288 patients (30 in pre-intervention period and 6489 in post-intervention period)	Educational meetings + educational materials + patient-mediated interventions vs. no intervention	**Non-indicated radiographs (post-intervention data) (no pre-intervention data) (n not reported)** 4.9%
OKR	Single-arm trial	Atkinson (2004)	Emergency department of a district general hospital, Luton	130 patients (58 in pre-intervention period and 72 in post-intervention period)	Reminders vs. no intervention	**Indicated radiographs (pre vs. post)** 24/28 (86%) vs. 36/37 (97%) (*p*-value not provided) **Non-indicated radiographs (pre vs. post)** 10/30 (33%) vs. 4/35 (11%), *p* = 0.016
		O’Sullivan (2006)	Emergency Department in Cork	79 patients (43 in pre-intervention period and 36 in post-intervention period)	Educational meetings + reminders vs. no intervention	**Indicated radiographs (pre vs. post)** 10/11 (91%) vs. 14/15 (93%) (p-value not provided) **Non-indicated radiographs (pre vs. post)** 19/32 (59%) vs. 9/21 (43%), *p* = 0.05
NEXUS	Single-arm trial	Rostas (2015)	Combined adult and pediatric Level I trauma centre, Richmond, Virginia	233 patients (128 in pre-intervention period and 105 in post-intervention period)	Clinical practice guideline vs. no intervention	**Indicated CT (pre vs. post)** 46/76 (61%) vs. 24/64 (38%), *p* = 0.01) **Non-indicated CT (pre vs. post)** 9/51 (18%) vs. 6/41 (15%) (*p*-value not provided)
		Baker (2020)	Emergency department	445 patients (42 in pre-intervention period and 403 in post-intervention period)	Educational meetings and materials + reminders + audit and feedback vs. no intervention	**Indicated radiographs (pre vs. post)** 23/42 (55%) vs. 313/403 (78%) (*p*-value not provided) **Non-indicated radiographs (pre vs. post)** 19/42 (45%) vs. 90/403 (22%), *p* = 0.002
		Griffith (2014)^d^	Level 1 adult trauma centre, Detroit, Michigan	895 patients (507 in pre-intervention period and 388 in post-intervention period)	Interactive educational Meeting + audit and feedback and continuous quality improvement + reminders vs. no intervention	**Indicated radiographs (pre vs. post)** 426/507 (84%) vs. 339/388 (87%) (*p*-value not provided) **Non-indicated radiographs (pre vs. post)** 81/507 (16%) vs. 49/376 (13%), *p* = 0.2
CCR	Single-arm trial	Griffith (2014)^d^	Level 1 adult trauma centre, Detroit, Michigan	895 patients (507 in pre-intervention period and 388 in post-intervention period)	Interactive educational meeting + audit and feedback and continuous quality improvement + reminders vs. no intervention	**Indicated radiographs (pre vs. post)** 297/416 (71%) vs. 249/320 (78%) (*p*-value not provided) **Non-indicated radiographs (pre vs. post)** 119/416 (29%) vs. 71/312 (23%) (*p*-value not provided)
**Overall imaging use**						
OAR	RCT	Tajmir (2017)^a^	Urgent care affiliated with quaternary care, academic hospital, Boston	613 patients (258 in pre-intervention period and 374 in post-intervention period)	Reminders vs. no intervention	**Radiography use (control vs. intervention)** **Ankle** 183/374 (49%) vs. 166/258 (64%), *p* < 0.01 **Foot** 202/374 (54%) vs. 141/258 (55%), *p* = 0.95 **Both ankle and foot** 59/374 (16%) vs. 65/258 (25%), *p* = 0.0039
		Auleley (1997)	Emergency departments of 5 Paris University teaching hospitals	4129 patients (1992 in the intervention and 2137 in the control group)	Educational meetings + educational materials vs. no intervention	**Radiography use (control vs. intervention)** **Pre-intervention** 1115/1132 (99%) vs. 1064/1086 (98%) (p-value not provided) **Intervention** 996/1005 (99%) vs. 691/906 (76%) (p-value not provided) **Post-intervention**^**c**^ No control data reported vs. 707/851 (83.1%) (p-value not provided)
	Quasi experimental controlled trial	Holroyd (2004)	Emergency departments of 4 major hospitals in Edmonton	6398 patients (3041 in the intervention and 3367 in the control group)	Int 1: Educational materials + interactive educational meetings Int 2: Educational meetings + Clinical Practice Guidelines Int 3: Educational meetings + audit and feedback + monitoring the performance of the delivery of healthcare VS. Int 1: Educational materials + interactive educational meetings	**Radiography use (control vs. intervention)** **Baseline period** (p-value not provided) - Total x-rays taken 571/623 (92%) vs. 444/481 (92%) - Dual x-ray, both ankle and foot 91/571 (16%) vs. 97/444 (22%) - Single x-ray, ankle, or foot 480/571 (84%) vs. 347/444 (78%) **End of Intervention 1** (*p*-value not provided) - Total x-rays taken 884/961 (92%) vs. 786/847 (93%) - Dual x-ray, both ankle and foot 133/884 (15%) vs. 131/786 (17%) - Single x-ray, ankle, or foot 751/884 (85%) vs. 655/786 (83%) **End of Intervention 2** (p-value not provided) - Total x-rays taken 697/760 (92%) vs. 676/719 (94%) - Dual x-ray, both ankle and foot 129/697 (19%) vs. 112/676 (17%) - Single x-ray, ankle, or foot 568/697 (75%) vs. 564/676 (78%) **End of Intervention 3** - Total x-rays taken 925/1023 (90%) vs. 919/984 (93%) - Dual x-ray, both ankle and foot 162/925 (18%) vs. 169/919 (18%) - Single x-ray, ankle, or foot 763/925 (75%) vs. 750/919 (76%)
	Single-arm trial	Silveira (2016)	Emergency Department, 793-bed, quaternary care, academic hospital, Boston	460 ED visits for 457 patients (205 in pre-intervention period and 255 in post-intervention period)	Reminders vs. no intervention	**Radiography use (pre vs. post**) **Ankle** 160/205 (78%) vs. 197/255 (77%), *p* = 0.839 **Foot** 103/205 (50%) vs. 117/255 (46%), *p* = 0.352 **Ankle or foot** 198/205 (97%) vs. 242/255 (95%), *p* = 0.379
		Ashurst (2014)	One tertiary care centre & one urban Emergency department in Bethlehem, Pennsylvania	60 patients (30 in pre-intervention period and 30 in post-intervention period)	Educational meetings + educational materials vs. no intervention	**Radiography use (pre- vs. post-intervention)** 27/30 (90%) vs. 24/30 (80%), *p* = 0.472
		Bessen (2009)	Emergency departments of a tertiary teaching hospital and a community hospital, Adelaide	1561 patients (459 in pre-intervention period and 1102 in post-intervention period)	Educational meetings + local consensus processes + reminders + audit and feedback vs. no intervention	**Radiography use (pre- vs. post-intervention)** **Tertiary hospital** 206/215 (96%) vs. 709/813 (87%), *p* < 0.001 **Community hospital** 223/244 (91%) vs. 228/289 (79%), *p* < 0.001
		Cameron (1999)	63 Ontario hospitals, including 5 primarily smaller community hospitals (Group A), 5 primarily larger community hospitals (Group B), 4 larger community hospitals and 1 teaching hospital (Group C)	407 clinicians seeing 1648 patients (830 in pre-intervention period and 818 in post-intervention period)	Educational meetings + educational materials vs. no intervention	**Radiography use (pre- vs. post-intervention)** **Group A hospitals** Ankle 119/162 (73%) vs. 141/190 (75%), *p* = 0.81 Foot 25/162 (15%) vs. 38/190 (20%), *p* = 0.27 **Group B hospitals** Ankle 176/241 (73%) vs. 190/235 (81%), *p* = 0.050 Foot *n* = 45/241 (19%) vs. 52/235 (22%), *p* = 0.20 **Group C hospitals** Ankle 181/240 (75% ) vs. 136/208 (65%), *p* = 0.022 Foot 46/240 (19%) vs. 44/208 (21%), *p* = 0.64
		Gwilym (2003)	Emergency department, Northampton General Hospital, Northampton	207 patients (106 in pre-intervention period and 101 in post-intervention period)	Educational meetings + educational materials vs. no intervention	**Radiography use (pre- vs. post-intervention)** 75/106 (71%) vs. 57/101 (56%), *p* < 0.05
		Stiell (1995)	Emergency departments across several regions	1,276,288 patients (30 in pre-intervention period and 6489 in post-intervention period)	Educational meetings + educational materials + patient-mediated interventions vs. no intervention	**Radiography use (pre- vs. post-intervention)** 5207/6288 (83%) vs. 3955/6489 (61%), *p* < 0.001
OKR	Single-arm trial	Atkinson (2004)	Emergency department of a district general hospital, Luton	130 patients (58 in pre-intervention period and 72 in post-intervention period)	Reminders vs. no intervention	**Radiography use (pre vs. post)** 34/58 (59%) vs. 40/72 (56%), *p* = 0.726
		O’Sullivan (2006)	Emergency Department in Cork	79 patients (43 in pre-intervention period and 36 in post-intervention period)	Educational meetings + reminders vs. no intervention	**Radiography use (pre- vs. post-intervention)** 29/43 (67%) vs. 23/36 (64%), *p* = 0.05
		Wigder (1999)	Emergency Department, Lutheran General Hospital, Chicago, Illinois	27 physicians seeing 362 patients (171 in pre-intervention period and 191 in post-intervention period)	Educational meetings + audit and feedback + educational materials + reminders vs. no intervention	**Radiography use (pre- vs. post-intervention)** 157/171 (92%) vs. 135/191 (71%) (p-value not provided)
NEXUS	Single-arm trial	Rostas (2015)	Combined adult and pediatric Level I trauma centre, Richmond, Virginia	233 patients (128 in pre-intervention period and 105 in post-intervention period)	Clinical practice guideline vs. no intervention	**CT use (pre vs. post**) 55/128 (43%) vs. 30/105 (29%), *p* = 0.01
CCR	Single-arm trial	Kerr (2005)	Emergency Department of a community, teaching hospital in Melbourne	211 patients with head and neck injury (98 in pre-intervention period and 113 in post-intervention period)	Educational meetings + reminders + Clinical practice guideline vs. no intervention	**Radiography use (pre- vs. post-intervention)** 66/98 (67.3%) vs. 57/113 (50.4%), *p* = 0.0187
CCHR	RCT	Stiell (2010)	12 hospital emergency department sites in the Canadian provinces of Ontario, Alberta, and British Columbia (6 teaching hospitals and 6 community hospitals)	4531 patients (2580 in intervention group and 1951 in control group)	Clinical practice guidelines + educational materials + educational meetings + reminders vs. no intervention	**CT use (control vs. intervention)** **Pre-intervention** 591/876 (68%) vs. 659/1049 (63%) (p-value not provided) **Post-intervention** 797/1075 (74%) vs. 1167/1531 (76%) (p-value not provided)
		Andruchow (2021)	Five urban acute care sites Calgary, Alberta: - one level one trauma centre, - three community EDs and - one urgent care centre	5687 patients (3085 in intervention group and 2602 in control group)	Reminders + audit and feedback or continuous quality improvement vs. no intervention	**CT use (pre- vs. post-intervention)** **Intervention** 2133/5136 (41.5%) vs. 1227/3085 (39.8%), *p* = 0.31 **Pre-intervention** 1979/4614 (42.9%) vs. 1112/2602 (42.7%) p-value not provided)
	Single-arm trial	Kim (2021)	Emergency Department of a large Midwestern academic medical centre in California	697 adult patients (467 in pre-intervention period and 230 in post-intervention period)	Educational meetings + education materials + reminders vs. no intervention	**Radiography use (pre- vs. post-intervention)** 399/467 (85.4%) vs. 169/230 (73.4%) (p-value not provided)
**Mean knowledge scores of the rules**						
OAR	RCT	Gravel (2010)	A paediatric ED located in an urban, tertiary care, Level 1 trauma centre, Montreal, Quebec	190 clinicians (95 in the control group and 95 in the intervention group) Pre-intervention, *N* = 190 Control group *N* = 95 Intervention group *N* = 95 At 3 weeks, *N* = 181 Control group *N* = 92 Intervention group *N* = 89 At 5 to 9 months, *n* = 138 Control group *N* = 68 Intervention group *N* = 70	Clinical practice guideline vs. standard description of OAR.	**Mean questionnaire scores for knowledge of rules, 0–13 for Ankle Rules and 0–10 for Foot Rules (a higher score means better knowledge) (Control vs. intervention)** **Preintervention** - Ankle rules 3.8 (95% CI: 3.0 to 4.6) vs. 3.5 (95% CI: 2.8 to 4.2) - Foot rules 2.4 (95% CI: 1.8 to 3.0) vs. 2.3 (95% CI: 1.7 to 2.9) **At 3 weeks** - Ankle rules 10.2 (95% CI: 9.6 to 10.9) vs. 10.9 (95% CI: 10.3 to 11.6) - Foot rules 7.5 (95% CI: 6.9 to 8.0) vs. 7.6 (95% CI: 7.0 to 8.1) **At 5 to 9 months** - Ankle rules 8.9 (95% CI: 8.3 to 9.5) vs. 10.1 (95% CI: 9.5 to 10.6) - Foot rules 6.5 (95% CI: 5.9 to 7.1) vs. 7.8 (95% CI: 7.2 to 7.3)
CCHR	Single-arm trial	Zakhari (2016)	Acute Care Academic Facility, New York	100 clinicians	Interactive educational meetings + reminders vs. no intervention	**Mean knowledge scores of CCHR, 0-100% (pre- vs. post-intervention)** 49% vs. 89% (p-value not provided)

*CCHR *Canadian *CT *Head Rule, *CCR *Canadian C-Spine Rule, *CCR *Cervical-Spine Rule, *CT *Computed Tomography, *ED *Emergency Department, *OAR *Ottawa Ankle Rules, *OKR *Ottawa Knee Rule, *RCT *Randomised Controlled Trial

^a^no baseline data for control and intervention period reported

^b^no intervention and control data for baseline reported and no control data for post-intervention period reported

^c^no control data for post-intervention period reported

^d^the sample size and results for the pre-intervention period are taken from the study Griffith (2013)

One single-arm trial found pop-up reminders in electronic medical records and reminder posters increased use of the Ottawa Ankle Rules (pre-to-post intervention: 56–96%, *p* < 0.001) among ED physicians [20]. One single-arm trial found educational meetings combined with educational materials increased use of the Ottawa Ankle Rules both before triage (pre-to-post intervention: 3–7%, *p* < 0.001) and after triage (20–83%, *p* < 0.001) among triage nurses, other nursing staff, residents, physician assistants, nurse practitioners, and physicians [10]. Another single-arm trial testing the same implementation strategy plus patient-mediated interventions reported that 95% of ED physicians used the Ottawa Ankle Rules during the post-intervention phase (no pre-intervention data was reported) [21]. One single-arm trial found educational meetings combined local opinion leaders, local consensus processes and reminder posters increased ED clinicians’ use of the Ottawa Ankle Rules in both tertiary (request forms: 41–83%, *p* < 0.001; case notes: 58–95%, *p* < 0.001) and community hospitals (request forms: 34–65%, *p* < 0.001; case notes: 52–81%, *p* < 0.001) [1].

#### Ottawa Knee Rule

One single-arm trial found pop-up reminders in electronic medical records and reminder posters had unclear effects on the use of the Ottawa Knee Rule (76–93%, *p*-value not reported) [22] among ED junior doctors. However, another single-arm trial found educational meetings combined with reminder posters increased use of the Ottawa Knee Rule (36–61%, *p* = 0.05) among ED non-consultant hospital doctors [23] (Table 4).

#### NEXUS

A single-arm trial had an unclear effect of distribution of NEXUS guidelines among paediatric ED physicians (85–80%, p-value not reported) [24] (Table 4).

#### Canadian CT Head Rule

An RCT compared the effectiveness of educational materials related to the Canadian CT Head Rule combined with pop-up reminders in electronic medical records and clinical practice guidelines against no intervention [25]. Following the intervention, the use of rules in the intervention group was reported as 78%. However, the study lacked data on the control group’s use of rules (Table 4). A single-arm trial found educational meetings combined with reminder posters and clinical practice guidelines related to the Canadian CT Head Rule reported a significant increase in the use of the rules by clinicians in the emergency department (pre-to-post intervention: 64.6–74.3%, *p* = 0.01) [26].

### Indicated and non-indicated imaging (secondary outcome)

#### Ottawa Ankle Rules

One RCT comparing educational meetings combined with educational materials related to the Ottawa Ankle Rules to no intervention found the indicated radiographs to be 98.5% and non-indicated radiographs to be 20.5% in the intervention group [16]. This study, however, was missing control group data for both indicated and non-indicated radiographs.

#### Ottawa Knee Rule

A single-arm trial found that use of reminder posters of Ottawa Knee rule had an unclear effect on use of indicated knee radiography (86–97%, *p*-value not provided) however, decreased use of non-indicated knee radiography (33–11%, *p* = 0.016) among ED junior doctors’ [22]. A single-arm trial studied the effect of educational meetings combined with reminders on Ottawa Knee rule use and found unclear effects on indicated imaging (91–93%, *p*-value not provided) and decreased non-indicated imaging (59–43%, *p* = 0.05) among the non-consultant hospital doctors [23] (Table 4).

#### NEXUS

One single-arm trial found that distribution of clinical practice guidelines related to NEXUS decreased paediatric ED physicians’ use of indicated imaging (pre-to-post intervention: 61–38%, *p* = 0.01) and however, had an unclear effect on non-indicated (18–15%, p-value not provided) cervical spine CT scans [24]. Another single-arm trial combining educational meetings, materials, and reminders had unclear effects on indicated cervical imaging (55–78%, *p*-value not provided) but decreased non-indicated imaging (45–22%, *p* = 0.002) [27]. Another single-arm trial of ED clinicians showed that interactive educational meetings combined with audit and feedback, continuous quality improvement and reminder posters had unclear effects on indicated radiographs (84–87%, p-value not reported) and no effect on non-indicated imaging (16–13%, *p* = 0.2) [28].

#### Canadian C-spine Rule

The same single-arm trial of ED clinicians involving the above multicomponent interventions about the Canadian C-spine Rule had unclear effects on both indicated (71–78%) and non-indicated imaging (29–23%) [28].

### Overall imaging use (secondary outcome)

#### Ottawa Ankle Rules

One RCT found that pop-up reminders about the Ottawa Ankle Rules in electronic medical records increased medical doctors’ and physician assistants’ use of radiography for ankle injuries compared to no intervention (64% vs. 49%, *p* < 0.01) [19]. Another RCT found educational meetings combined with educational materials related to the Ottawa Ankle Rules reduced ED physicians’ use of ankle radiography (pre-to-post intervention: 98% vs. 76%, *p* = 0.03, relative reduction = 22.4%, 95% confidence interval [CI], 19.8-24.9%) whereas there was no decrease in the no intervention control group (99% vs. 99%, relative increase = 0.5%, 95% CI: 0-1.4%, *p*-value not reported) [16]. A quasi-experimental controlled trial found a combination of three interventions at different time points (e.g., educational materials and meetings initially, then guidelines – Ottawa Ankle Rules, then audit and feedback) did not affect ED physicians’ use of ankle radiography compared to no intervention (92% vs. 93%, *p* = 0.54) [29].

One single-arm trial found that reminders about the Ottawa Ankle Rules did not affect clinicians’ use of ankle/foot radiography (ankle - pre vs. post: 78% vs. 77%, *p* = 0.839; foot − 50% vs. 46%, *p* = 0.352; ankle and foot − 97% vs. 95%, *p* = 0.379) [20] (Table 4). Five single-arm trials studied the effect of educational meetings combined with other interventions [10] such as educational materials [10, 30–32] local consensus processes [1], reminders [1] and patient-mediated interventions [21] on clinicians’ use of ankle imaging. Some studies found a decrease in clinicians’ (ranging from 9% [1] to 22% [21]) use of ankle radiography whereas, one study did not find a significant change in ankle radiography referrals (73% v. 78%, *p* = 0.11) [31].

#### Ottawa Knee Rule

A single-arm trial found that reminders about the Ottawa Knee rule did not affect clinicians’ use of knee radiography (59% vs. 56%, *p* = 0.726) [22] (Table 4). Two other single-arm trials found that educational meetings when combined with reminders [23], audit and feedback [33] and educational materials [33] related to Ottawa Knee Rule found a reduction in non-consultant hospital doctors’ (67% vs. 64%, *p* = 0.05) [23] use of knee radiography whereas there was an unclear effect on ED physicians’ (92% vs. 71%, *p*-value not provided) [33] use of knee radiography.

#### NEXUS

One single-arm trial found that the distribution of NEXUS guidelines decreased paediatric ED physicians’ use of cervical spine CT scans (pre-to-post intervention: 43–29%, *p* = 0.01) [24] (Table 4).

#### Canadian C-spine Rule

A single-arm trial investigated the impact of combining educational meetings, and reminders with clinical practice guidelines on the Canadian C-Spine Rule [34] and observed a non-statistical reduction in c-spine imaging (pre vs. post: 67.3% vs. 50.4%, *p* = 0.16) [34].

#### Canadian CT Head Rule

An RCT found clinical practice guidelines combined with educational meetings, educational materials and pop-up reminders about the Canadian CT Head rule in electronic medical records increased ED physicians’ use of CT imaging for head injuries in both the intervention (63% vs. 76%, difference = 13.3%, 95% CI: 9.7-17%) and the control groups (68% vs. 74%, difference = 6.7%, 95% CI: 2.6%-0.8%) but the between-group difference was not significant (*p* = 0.16) [25]. Another RCT that combined reminders about the Canadian CT Head Rule with audit and feedback and continuous quality improvement showed no decrease in the use of head CT among clinicians, in both the intervention (pre vs. post use: 41.5% vs. 39.8%, *p* = 0.31) and the control group (42.9% vs. 42.7%, p-value not provided) [17] when compared with no intervention. A single-arm trial found that a combination of educational meetings and reminders with clinical practice guidelines on Canadian CT Head Rule had unclear effects on head CT (83.4% to 73.4, p-value not provided) [35].

### Mean knowledge scores (secondary outcome)

#### Ottawa Ankle Rules

One RCT found the distribution of the Ottawa Ankle Rules using a mnemonic and the standard description of the rules was not superior to a standard description of the rules alone for increasing residents’ and medical students’ knowledge of the ankle component (mean = 10.9 vs. 10.2 on a 0–13 scale, difference = 0.7, 95% CI: -0.3-1.7, *p* = 0.16) and foot component of the rules (7.6 vs. 7.5, difference = 0.1, 95% CI: -0.7-0.9, *p* = 0.80) at three weeks post-intervention [18]. At 5–9 months, there was no effect on knowledge of the ankle component (10.1 vs. 8.9; difference = 1.18; 95% CI: 0.57–1.81, *p* = 0.039) but an increase in mean knowledge of the foot component of the rules (6.5 vs. 7.8; mean difference = 1.32; 95% CI: 0.78–1.87, *p* = 0.004) [18] (Table 4).

#### Canadian CT Head Rule

One single-arm trial found interactive educational meetings and materials of the Canadian CT Head rule had unclear effects on clinicians’ knowledge score (scale range 0-100%) of the rule (mean of 49% to mean of 89%, no p-value reported) [36]. Clinicians included nurse practitioners, physician assistants, attending physicians, postgraduate students and registered nurses.

## Discussion

Results from the four RCTs that provided appropriate data for some outcomes in this review are conflicting. One trial suggested reminders may increase medical doctors’ and physician assistants’ use of the Ottawa Ankle Rules but could also lead to an increase in ankle imaging [19]. Another trial suggested educational meetings and materials on Ottawa Ankle Rules may decrease ED physicians’ use of imaging for ankle injuries [16]. Regarding the Canadian CT Head Rule, the effectiveness of reminders when combined with audit and feedback, did not significantly reduce head CT use among clinicians [17]. However, another trial found that educational meetings and materials on the Canadian CT Head rule, when combined with the distribution of clinical practice guidelines and reminders, may *increase* ED physicians’ use of CT imaging for head injuries [25]. Unfortunately, none of these RCTs provided relevant data on use of indicated and non-indicated imaging.

For knowledge of the rules, one RCT found clinical practice guidelines did not improve clinicians’ short-term knowledge of the Ottawa Ankle Rules but may increase long-term knowledge [18]. Results from 16 single-arm trials suggested that multi-component interventions may increase clinicians’ use of decision rules and knowledge but their impact on imaging use (overall, indicated and non-indicated) is mixed.

### Strengths and weaknesses of the study

This review has several strengths including a comprehensive search strategy across multiple databases to identify eligible studies, a large sample size (*n* = 1271 clinicians and *n* = 35,010 patients) and several methodological steps performed in duplicate (e.g., selection of studies, data extraction, risk of bias assessment) to improve validity and accuracy. There are also some limitations. All the included studies were conducted in developed countries (USA, Canada, UK, Australia, France and Ireland), which may limit the generalisability of the findings to developing countries. We were unable to perform a meta-analysis due to heterogeneity in interventions and outcomes. Most studies were at high risk of bias as they did not have a control group, did not use randomisation, did not blind participants which may lead to Hawthorne effect, and did not report data appropriately. These limitations may introduce bias, affecting result reliability. Hence, care should be taken when interpreting findings.

### Meaning of the study

The effectiveness of implementation strategies varied across studies, particularly when certain strategies were combined with others. For example, while educational meetings and materials related to the Ottawa Ankle Rules seem to decrease the use of imaging (overall) for ankle injuries [16], the combination of educational meetings, materials, clinical practice guidelines, and reminders regarding the Canadian CT Head rule increased the utilisation of CT (overall) for head injuries [25]. Variations in the apparent effectiveness of some implementation strategies could be due to differences in the acceptability of rules among clinicians. Our recent systematic review (34 studies) explored awareness and use of the same five validated imaging decision rules among clinicians (Canadian CT Head Rule, Canadian C-spine Rule, NEXUS guidelines, Ottawa Ankle Rule, and Ottawa Knee Rule) [14]. We found varying levels of usage for different rules and that clinicians’ attitudes towards these rules may contribute to the differences in their utilisation. For example, some clinicians easily accept some of the rules, while others don’t use the rules or don’t plan on using them in the future.

Variation in the effectiveness of some implementation strategies could also be attributed to barriers to implementation experienced by clinicians, which our previous review provided insights on [14]. Our review highlighted that a substantial number of clinicians lack awareness of validated imaging decision rules with percentages ranging from 31 to 99% across different regions and rules. The review also found that even among those who are aware of these rules, there appears to be a gap in their implementation. Some of the most commonly reported barriers to using imaging decision rules included lack of research to support their use (64%) [37], the complexity of the rules (63%) [38], lack of time at triage to use the rules or EDs being too busy (39%), and heavy workload making it difficult to apply the rules (37%) [39]. This suggests there is a need to identify and tailor implementation strategies to address these barriers to achieve the desired change in clinicians’ use of decision rules [40].

Categorising barriers into factors related to individual clinicians, social context, and organisation could be one way to guide the development of implementation strategies that increase clinicians’ adoption of decision rules [1]. Grimshaw (2001) summarised 41 systematic reviews focusing on professional education and quality assurance interventions to improve quality of care. They found quantitative data suggesting that using a variety of interventions targeting multiple barriers to change is more likely to result in behaviour change compared to relying on a single intervention [41]. However, it should also be noted that although multi-component interventions may be more comprehensive and have the potential to address multiple barriers to implementation, it is important to consider the potential costs and resources required for implementing multi-component interventions [42].

### Comparison to existing research

Reminders were seen to decrease the use of non-indicated imaging for ankle and knee injuries when used as a single-component intervention [22, 23] or in combination with educational meetings [22, 23]. These findings appear to be consistent with the effectiveness of reminders in the broader literature. For example, an overview of 41 systematic reviews that aimed to synthesise interventions to improve the quality of care provided by clinicians found that reminders were more effective than other interventions in changing clinicians’ behaviour [41]. Reminders prompt adherence to clinical guidelines by providing simple, timely information aimed at improving professional practices and patient outcomes [43], and may be particularly useful for busy clinicians who treat patients with a range of conditions (e.g. general practitioners, ED physicians).

A study among paediatric patients showed a non-significant decrease in ED physicians’ use of NEXUS guidelines after the distribution of clinical practice guidelines [24]. However, there was also a notable reduction in neck CT scans, representing a positive outcome. The findings align with the existing literature. For example, a systematic review of 4 studies involving 4502 paediatric patients highlighted the low sensitivity, reliability, and clinical acceptability of the NEXUS criteria in pediatric trauma patients [44]. Furthermore, uncertainties in evaluating mental status in children under 3 years old [45] might also contribute to the observed reduction in NEXUS guideline adherence among paediatric ED physicians.

Our review demonstrated that the effectiveness of educational interventions for reducing clinicians’ use of imaging (overall) may depend on the specific context and type of imaging. One RCT found that educational meetings and educational materials related to Ottawa Ankle Rules may decrease clinicians’ use of ankle radiography (overall), whereas, another RCT found that the addition of reminders of Canadian CT Head rule use and clinical practice guidelines to these interventions may increase ED physicians’ use of CT (overall) for head injuries [25]. This appears to be consistent with the broader literature. For example, a systematic review (*n* = 11 RCTs) evaluated the effectiveness of knowledge translation interventions in enhancing the adoption and implementation of clinical practice guidelines for musculoskeletal conditions [46]. The review found that there were inconsistent effects of the interventions on professional practice (such as a change in practice or behaviour, knowledge, skills, and self-efficacy) [46]. The study found that while educational meetings had a positive effect in enhancing the appropriate use of diagnostic imaging for spinal disorders, combining interactive educational meetings with local opinion leaders did not significantly impact physiotherapists’ clinical practice for non-specific low back pain. Another systematic review (*n* = 5 studies) studied the effect of multi-component interventions such as interactive educational meetings (which included interactive sessions, practical sessions, problem-solving, feedback, and reminders) on clinicians’ implementation of certain guideline recommendations from low back pain and whiplash [47]. The study highlighted the importance of considering the quality and quantity of strategies when implementing any physiotherapy guidelines, as studies with higher guideline adherence at baseline, or those that used multiple educational meetings achieved greater adherence compared to others.

### Unanswered questions and future research

While we found some evidence on the effectiveness of implementation strategies for increasing clinicians’ use of imaging decision rules, there is still a need for high-quality RCTs in this area. For example, of the 22 included studies, only five were RCTs and only one of these reported appropriate data for our primary outcome. Similarly, of the four RCTs that reported data on imaging use, only one reported on whether the imaging was indicated or not, but the study did not report data from the control group, making it hard to draw conclusions about the effectiveness of implementation strategies for these outcomes. Another area for future research is to compare different combinations of implementation strategies to see if using multiple strategies together is more effective than using a single strategy. Additionally, there should be a focus on understanding whether different types of imaging decision rules require different implementation strategies for effective uptake. This could be investigated by exploring the effectiveness of different types of multi-component interventions such as a combination of reminders, educational meetings, and educational materials, tailored to the specific needs of different healthcare professionals, as well as evaluating the sustainability of implementing these strategies over time.

## Conclusion

Reminders whether as a single-component intervention or as part of a multi-component approach when combined with educational meetings have demonstrated effectiveness in increasing clinicians’ use of the Ottawa Ankle Rules and reducing ankle imaging. Pop-up reminders, meetings, and posters improve adherence to the Ottawa Knee Rule and Canadian CT Head Rule. However, the dissemination of the NEXUS guidelines slightly reduced the use of the rule but notably also reduced the use of neck CT among paediatric ED physicians representing a positive outcome. These varying effects of interventions highlight the need for future research to compare different combinations of implementation strategies as this will help explore whether specific imaging decision rules benefit from tailored implementation approaches. A lack of appropriately conducted and reported RCTs makes it difficult to draw firm conclusions.

### Supplementary Information

**Supplementary Material 1.**

## Data Availability

All data generated or analysed during this study are included in this published article (and its supplementary information files).
