# Supplementary material for: Effectiveness of implementation strategies for increasing clinicians’ use of five validated imaging decision rules for musculoskeletal injuries: a systematic review
Source: BMC Emerg Med. 2024 May 17;24:84. doi: 10.1186/s12873-024-00996-x (PMC11100091; doi:10.1186/s12873-024-00996-x)
Supplement: Supplementary file 1 — Supplementary Material 1. [file 12873_2024_996_MOESM1_ESM.docx]

**Supplementary Tables**

Supplementary File 1: Search strategy

Supplementary Table 1: Standardised data extraction form

Supplementary Table 2: Characteristics of all the included studies.

Supplementary Table 3: Risk of bias for each study as per the Cochrane EPOC risk-of-bias tool

**Supplementary File 1: Search strategy**

**Ovid MEDLINE(R) ALL 1946 to March 11, 2024**

1. Ott#wa adj3 rule*.mp
2. Ankle rule*.mp
3. Ott#wa ankle guideline*.mp
4. Ott#wa foot and ankle rule*.mp
5. Knee rule*.mp
6. (Canadian adj3 rule*).mp
7. C-spine rule*.mp
8. Cervical-spine rule*.mp
9. Spine rule*.mp
10. nexus guideline*.mp
11. national emergency x-radiography utili#ation stud*.mp
12. (nexus adj3 criteria*).mp
13. ("nexus" and "guideline*").mp
14. Canadian Computed Tomography head rule*.mp.
15. Head rule*.mp
16. 1 or 2 or 3 or 4 or 5 or 6 or 7 or 8 or 9 or 10 or 11 or 12 or 13 or 14 or 15
17. limit 16 to humans

**Embase Classic 1947 to 1973 and Embase 1974 to March 11, 2024**

1. (Ott#wa adj3 rule*).mp.
2. Ankle rule*.mp.
3. (Ott#wa adj3 guideline*).mp.
4. (Ott#wa foot and ankle rule*).mp. [mp=title, abstract, heading word, drug trade name, original title, device manufacturer, drug manufacturer, device trade name, keyword, floating subheading word, candidate term word]
5. Knee rule*.mp.
6. (Canadian adj3 rule*).mp.
7. C-spine rule*.mp.
8. Cervical-spine rule*.mp.
9. Spine rule*.mp.
10. nexus guideline*.mp.
11. national emergency x-radiography utili#ation stud*.mp.
12. (nexus adj3 criteria*).mp.
13. ("nexus" and "guideline*").mp.
14. Canadian Computed Tomography head rule*.mp.
15. Head rule*.mp.
16. 1 or 2 or 3 or 4 or 5 or 6 or 7 or 8 or 9 or 10 or 11 or 12 or 13 or 14 or 15
17. limit 16 to humans

**Cochrane Central Register of Controlled Trials March 11, 2024**

1. (Ott#wa adj3 rule*).mp.
2. Ankle rule*.mp.
3. (Ott#wa adj3 guideline*).mp.
4. (Ott#wa foot and ankle rule*).mp. [mp=title, original title, abstract, mesh headings, heading words, keyword]
5. Knee rule*.mp.
6. (Canadian adj3 rule*).mp.
7. C-spine rule*.mp.
8. Cervical-spine rule*.mp.
9. Spine rule*.mp.
10. national emergency x-radiography utili#ation stud*.mp.
11. (nexus adj3 criteria*).mp.
12. ("nexus" and "guideline*").mp.
13. Head rule*.mp.
14. 1 or 2 or 3 or 4 or 5 or 6 or 7 or 8 or 9 or 10 or 11 or 12 or 13

**Scopus search March 11, 2024**

1. Ott?wa w/3 rule*
2. “Ankle rule*”
3. “Ott?wa ankle guideline*”
4. “Ott?wa foot and ankle rule*”
5. “Knee rule*”
6. Canadian w/3 rule*
7. “C-spine rule*”
8. “Cervical-spine rule*”
9. “Spine rule*”
10. “nexus guideline*”
11. “national emergency x-radiography utili?ation stud*”
12. nexus w/3 criteria*
13. "nexus" and "guideline*"
14. “Canadian Computed Tomography head rule*”
15. “Head rule*”

( TITLE-ABS-KEY ( ott?wa W/3 rule* ) ) OR ( TITLE-ABS-KEY ( "Ankle rule*" ) ) OR ( TITLE-ABS-KEY ( "Ott?wa ankle guideline*" ) ) OR ( TITLE-ABS-KEY ( "Ott?wa foot and ankle rule*" ) ) OR ( TITLE-ABS-KEY ( "Knee rule*" ) ) OR ( TITLE-ABS-KEY ( canadian W/3 rule* ) ) OR ( TITLE-ABS-KEY ( "C-spine rule*" ) ) OR ( TITLE-ABS-KEY ( "Cervical-spine rule*" ) ) OR ( TITLE-ABS-KEY ( "Spine rule*" ) ) OR ( TITLE-ABS-KEY ( "nexus guideline*" ) ) OR ( TITLE-ABS-KEY ( "national emergency x-radiography utili?ation stud*" ) ) OR ( TITLE-ABS-KEY ( nexus W/3 criteria* ) ) OR ( TITLE-ABS-KEY ( nexus W/3 criteria* "nexus" AND "guideline*" ) ) OR ( TITLE-ABS-KEY ( "Canadian Computed Tomography head rule*" ) ) OR ( TITLE-ABS-KEY ( "Head rule*" ) )

**CINAHL via EBSCO March 11, 2024**

- 1. S1 "“Ott?wa w3 rule*”"
  2. S2 "“Ankle rule*”"
  3. S3 "“Ott?wa ankle guideline*”"
  4. S4 "“Ott?wa foot and ankle rule*”"
  5. S5 "“Knee rule*”"
  6. S6 ""Canadian w3 rule*""
  7. S7 "“C-spine rule*”"
  8. S8 "“Cervical-spine rule*”"
  9. S9 "“Spine rule*”"
  10. S10 "“nexus guideline*”"
  11. S11 "“national emergency x-radiography utili?ation stud*”"
  12. S12 "“nexus w3 criteria*”"
  13. S13 ""nexus" and "guideline*""
  14. S14 "“Canadian Computed Tomography head rule*”"
  15. S15 "“Head rule*”"
  16. S16 S1 OR S2 OR S3 OR S4 OR S5 OR S6 OR S7 OR S8 OR S9 OR S10 OR S11 OR S12 OR S13 OR S14 OR S15

**Web of Science Core Collection March 11, 2024**

1. (Ott?wa ankle rule*)
2. ("Ott?wa rule*")
3. (Ott?wa ankle guideline*)
4. (Ott?wa foot and ankle rule*)
5. ("knee rule*")
6. ("Ottawa knee rule*")
7. C-spine rule*
8. "C-spine rule*"
9. "Cervical-spine rule*"
10. “Spine rule*”
11. “nexus guideline*”
12. “national emergency x-radiography utili?ation stud*”
13. "nexus criteria*"
14. “Canadian Computed Tomography head rule*”
15. “Canadian CT head rule*”
16. “head rule*”

**Supplementary Table 1: Standardised data extraction form**

| **Study characteristics** | Study year |
| --- | --- |
|  | Citation |
|  | Study type |
|  | Country |
|  | Study site/ setting |
|  | Recruitment method |
|  | Participant demographics |
|  | Musculoskeletal injury area/condition |
|  | Decision rule investigated |
|  | Clinician types included |
|  | Sample size |
|  | Response rate |
|  | Inclusion criteria |
|  | Exclusion criteria |
|  | Objective |
|  | Intervention |
|  | Control |
|  | Randomisation |
|  | Data collection time points |
|  | Data collection method |
| **Pre-intervention outcome data for intervention and control group (NB: there may not be a control group. If not, leave this blank)** | Self-reported use |
|  | Documented use of the rules |
|  | Documentation of clinical features |
|  | Patients referred for radiography |
| **Post-intervention outcome data for intervention and control group (NB: there may not be a control group. If not, leave this blank)** | Self-reported use |
|  | Documented use of the rules |
|  | Documentation of clinical features |
|  | Patients referred for radiography |
| **Report these outcomes pre- and post, and for the control and intervention group if applicable** | Other outcomes |
|  | Knowledge of rules |
| **EPOC Cochrane risk of bias tool** | Random sequence generation |
|  | Allocation concealment |
|  | Baseline outcome measurements similar |
|  | Baseline characteristics similar |
|  | Incomplete outcome data |
|  | Knowledge of the allocated interventions adequately prevented during the study |
|  | Protection against contamination |
|  | Selective outcome reporting |
|  | Other risks of bias |

**Supplementary Table 2. Characteristics of all the included studies**.

| S No. | **Author (year), country, study design** | **Clinician types included** | **Intervention and control** | **Sample size and participants included** | **Demographics** | **Decision rule** |
| --- | --- | --- | --- | --- | --- | --- |
|  | Andruchow (2021)  **Country:** Canada  **Study design:** Randomised controlled trial | - Emergency physicians | Reminders + audit and feedback or continuous quality improvement vs no intervention | 104 physicians  5687 patients with head injury | **Patients**  **Post-intervention period**  **Control group N=2602**   - Age (median, IQR): 45 (28, 68) - Female: 1215 (46.7%)   **Intervention group N= 3085**   - Age (median, IQR): 44 (28, 66) - Female: 1498 (48.6%)   **Clinicians**  Number of clinicians N=104 | Canadian CT Head Rule |
|  | Ashurst (2014)  **Country:** USA  **Study design:** Single-arm trial | - Physician assistants - Emergency nurse practitioners - Emergency physicians - Junior doctors - Triage nurses - Other nursing staff | Educational meetings + educational materials vs no intervention | 60 patients: Adults (aged 18 years or older) presenting with an isolated traumatic ankle or foot injury with no other complaints or injuries present | **Patients**  Pre-intervention period N=30  Post-intervention period N=30  Overall N=60   - Mean age (SD): 36.5 (16.58) years - Female: 37 (61.7%)   **Clinicians**  Not reported | Ottawa ankle rules |
|  | Atkinson (2004)  **Country:** UK  **Study design:** Single-arm trial | - Junior doctors | Reminders vs no intervention | 130 patients: Adults (aged 18 years or older) presenting with a primary, isolated, non-penetrating knee injury | **Patients**  Pre-intervention period N=58  Post-intervention period N=72  **Clinicians**  Not reported | Ottawa knee rules |
|  | Auleley (1997)  **Country:** France  **Study design:** Randomised controlled trial | - Junior doctors - Emergency physicians | Educational meetings + educational materials vs no intervention | 4129 patients: Adults presenting with an ankle or mid-foot injury and referred to the emergency department within 10 days of injury | **Patients**  **Pre-intervention period**  **Control group N=1132**   - Mean age (range): 35 (18-92) years - Female: 512 (45%)   Treating physicians:   - House staff physicians: 740 (65.4%) - Emergency department physicians: 392 (34.5%)   **Intervention group N=1086**   - Mean age (range): 36 (18-91) years - Female: 535 (49%)   Treating physicians:   - House staff physicians: 647 (59.5%) - Emergency department physicians: 439 (40.5%)   **Intervention Period**  **Control group N=1005**   - Mean age (range): 34 (18-93) years - Female: 459 (46%)   Treating physicians:   - House staff physicians: 609 (60.6%) - Emergency department physicians: 396 (39.4%)   **Intervention group N=906**   - Mean age (range): 34 (18-94) years - Female: 443 (49%)   Treating physicians:   - House staff physicians: 443 (48.9%) - Emergency department physicians: 463 (51.1%)   **Clinicians**  Not reported | Ottawa ankle rules |
|  | Baker (2020)  **Country:** USA  **Study design:** Single-arm trial | - ED and imaging manager, - ED medical director, - Emergency physician, - Resident, - Emergency nurse practitioner, - Quality nurse | Educational meetings and materials + reminders + audit and feedback | Non-trauma ED patients over 18 years of age | **Patients**  Not reported  **Clinicians**  Not reported | NEXUS |
|  | Bessen (2009) **Country:** Australia  **Study design:** Single-arm trial | - Triage nurses - Emergency nurse practitioners - Medical staff | Educational meetings + local consensus processes + reminders vs no intervention | 377 clinicians  1561 patients:  Adults presenting with an ankle injury | **Patients**  Pre-intervention period N=459   - Tertiary: 215 (46.8%) - Community: 244 (53.2%)   Intervention period N=1102   - Tertiary: 813 (73.8%) - Community: 289 (26.2%)   **Clinicians**  Number of clinicians N=377   - Tertiary: 315 (83.6%)   Community: 62 (16.4%) | Ottawa ankle rules |
|  | Cameron (1999)  **Country:** Canada  **Study design:** Single-arm trial | - Emergency nurses - Family physicians - Other health care professionals (e.g. physiotherapists, x-ray technicians and managers) | Educational meetings + educational materials vs no intervention | 407 clinicians  1648 patients:  Adults (aged 18 years or older) presenting with an ankle injury caused by acute blunt trauma (including twisting, falling and direct blows) | **Patients**  Pre-intervention period N=830  Post-intervention period N=818  Group A **(little or no prior use of Ottawa Ankle Rules + educational intervention),** N=516   - Mean age (range): 39 years (18-91) - Female: 239 (46%) - Total ineligible: 164 (32%)   Group B **(some prior use of Ottawa Ankle Rules + educational intervention),** N=567   - Mean age (range): 37 years (18-88) - Female: 263 (46%) - Total ineligible: 91 (16%)   Group C **(active local implementation of Ottawa Ankle Rules, no educational intervention),** N=565   - Mean age (range): 37 years (18-90) - Female: 261 (46%) - Total ineligible: 117 (21%)   **Clinicians**  Not reported | Ottawa ankle rules |
|  | Gravel (2010)  **Country:** Canada  **Study design:** Randomised controlled trial | - Medical students - Speciality residents (family medicine, emergency medicine, paediatric, other) | Clinical practice guideline vs standard description of OAR. | 190 clinicians | **Patients**  Not reported  **Clinicians**  **Pre-intervention**  **Control group N=95**  Female: 74 (78%)  Medical students: 49 (51%)  Speciality residents: 46 (48%)   - Family medicine: 33 - Emergency medicine: 3 - Paediatric: 10   **Intervention group N=95**  Female: 72 (76%)  Medical students: 47 (49%)  Speciality residents: 48 (51%)   - Family medicine: 28 - Emergency medicine: 6 - Paediatric: 11   **Post-intervention**  **Control group N=68**  **Intervention group N=70** | Ottawa ankle rules |
|  | Griffith (2014)  **Country:** USA  **Study design:** Single-arm trial | - Emergency physicians - Junior doctors - Physician assistants | Interactive educational  meeting + audit and feedback or continuous quality improvement + reminders vs no intervention | 895 patients: Adults who underwent CT screening of the cervical spine as part of an evaluation for blunt trauma | **Patients**  **Phase 2 (pre-intervention period) N=507**   - Mean age (range) 44 (18-100): years - Female: 198 (39.1%)   **Mechanism of injury**   - Motor vehicle crashes: 203 (40%) - Falls: 150 (29.6%) - Assault: 99 (19.5%) - Pedestrian versus motor vehicle: 23 (4.5%) - Other/not recorded: 32 (6.4%)   **Phase 3 (post-intervention period) N=388**   - Mean age (range): 48 years (18-98) - Female: 171 (44.1%)   **Mechanism of injury**   - Motor vehicle crash: 119 (30.7%) - Falls: 142 (36.6%) - Assault: 8 (22.7%) - Pedestrian versus motor vehicle: 13 (3.4%) - Other/not recorded: 26 (6.7%)   **Clinicians**  Not reported | NEXUS and Canadian C-spine Rules |
|  | Gwilym (2003)  **Country:** UK  **Study design:** Single-arm trial | - Triage nurses - Medical staff | Educational meetings + educational materials vs no intervention | 207 patients: Adults aged 18-55 years old presenting with an isolated ankle injury, with no significant previous ankle injury | **Patients**  Before teaching N=106  After teaching N=101  **Clinicians**  Not reported | Ottawa ankle rules |
|  | Helms (2023)  **Country: USA**  **Study design:** Single-arm trial | - Triage nurses - Medical staff - Emergency physicians - Registered nurses, - Paramedics - Patient care technicians | Interactive educational meetings + educational materials + Clinical Practice Guidelines + reminder | 600 patients: Adults (aged 18 to 64 years) with traumatic brain injury | **Patients**  **Pre-intervention** **period** N=264  **Post-intervention** **period** N=336  **Clinicians**  Not reported | Canadian CT Head Rule |
|  | Holroyd (2004)  **Country:** Canada  **Study design:** Quasi-experimental controlled trial | - Emergency physicians | Int 1: Educational materials + interactive educational meetings  Int 2: Educational meetings + Clinical Practice Guidelines  Int 3: Educational meetings + audit and feedback + monitoring the performance of the delivery of healthcare  VS.  Int 1: Educational materials + interactive educational meetings | 6398 patients: Adults (aged 18 years and above) presenting with a traumatic injury of the ankle or midfoot | **Patients**  **Control group N=3367**  Royal Alexandra Hospital n=1936   - Mean age (SD): 37 (15) years - Female: 898 (46%)   Misericordia Hospital n=1431   - Mean age (SD): 37 (16) years - Female: 705 (49%)   **Intervention group N=3031**  University of Alberta Hospital n=1290   - Mean age (SD): 35 (15) years - Female: 636 (49%)   Grey Nuns Hospital n=1741   - Mean age (SD): 36 (14) years - Female: 824 (47%)   **Clinicians**  Not reported | Ottawa ankle rules |
|  | Kerr (2005)  **Country:** Australia  **Study design:** Single-arm trial | - Medical staff - Junior staff - ED nurses - Emergency physicians | Educational meetings + reminders + Clinical practice guidelines vs no intervention | 211 patients with head and neck injury | **Patients**  **Pre-Intervention** **period** N=98   - Median age (range): 33 (16 to 83) - Female: 36 (35.7%)   **Mechanism of injury**   - Motor Car Accident: 50 (51%) - Collision/struck by object and/or person: 21 (21.4%) - Fall: 13 (13.3%) - Motor Bike Accident: 8 (8.2%) - Pedal cyclist: 4 (4.1%) - Pedestrian: 3 (3.1%)   **Post-Intervention** **period** N=113   - Median age (range): 38 (16 to 87) - Female: 154 (60.4%) - Malleolar zone pain: 196 (76.9%) - Midfoot zone pain: 111 (43.5%)   **Mechanism of injury**   - Motor Car Accident: 62 (54.9%) - Collision/struck by object and/or person: 10 (8.8%) - Fall: 17 (15%) - Motor Bike Accident: 10 (8.8%) - Pedal cyclist: 7 (6.2%) - Pedestrian: 7 (6.2%)   **Clinicians**  Not reported | Canadian C-spine rule |
|  | Kim (2021)  **Country:** USA  **Study design:** Single-arm trial | - Emergency medicine resident physicians, - Attending physicians, and - Advanced practice registered nurses. | Educational meetings + education materials + reminders vs no intervention | 697 adult patients with minor head injury within 24 hours | **Patients**  **Pre-intervention** **period** N=467  **Post-intervention** **period** N=230  **Clinicians**  Not reported | Canadian CT Head Rule |
|  | O'Sullivan (2006)  **Country:** Ireland  **Study design:** Single-arm trial | - Emergency physicians | Educational meetings + reminders vs no intervention | 79 patients:  Adults presenting to the emergency department with an acute knee injury | **Patients**  **Pre-intervention** **period** N=43  **Post-intervention** **period** N=36  **Clinicians**  Not reported | Ottawa knee rules |
|  | Rostas (2015)  **Country:** USA  **Study design:** Single-arm trial | - Adult trauma surgeons - Emergency physicians | Clinical practice guideline vs no intervention | 233 patients: Children <15 years old. First-tier or second-tier trauma team alerts | **Patients**  **Pre-intervention** **period** N=128   - Median age (IQR): 6.0 (7.0) - Median Injury Severity Score (IQR): 4.0 (9.0)   **Post-intervention** **period** N=105   - Median age (IQR): 7.0 (7.0) - Median Injury Severity Score (IQR): 4.0 (8.0)   **Clinicians**  Not reported | NEXUS |
|  | Silveira (2016)  **Country:** USA  **Study design:** Single-arm trial | - Emergency physicians | Reminders vs no intervention | 460 ED visits for 457 patients: Adults (aged 18 years or older) presenting with an acute blunt ankle injury (within 10 days) | **Patient visits**  **Pre-intervention period** N=205   - Mean age (SD): 38.5 (15.9) - Female: 142 (69.3%) - Malleolar zone pain: 152 (74.2%) - Midfoot zone pain: 88 (42.9%)   **Mechanism of injury**   - Twist: 174 (84.9%) - Fall from height: 17 (8.3%) - Direct blow: 11 (5.4%) - MVA: 2 (1.0%) - Other: 1 (0.5%)   **Post-Intervention** **period** N=255   - Mean age (SD): 37.6 (15.9) - Female: 154 (60.4%) - Malleolar zone pain: 196 (76.9%) - Midfoot zone pain: 111 (43.5%)   **Mechanism of injury**   - Twist: 198 (77.7%) - Fall from height: 23 (9.0%) - Direct blow: 19 (7.5%) - MVA: 3 (1.2%) - Other: 12 (4.7%)   **Clinicians**  Not reported | Ottawa ankle rules |
|  | Stiell (1995)  **Country:** Canada  **Study design:** Single-arm trial | - Emergency physicians - Family physicians - Junior doctors | Educational meetings + educational materials + patient-mediated interventions vs no intervention | 12777 patients: Adults presenting with acute ankle trauma from any mechanism of injury | **Patients**  **Pre-intervention period** N=6288   - Median age (range): 32 (18-101) - Female: 2890 (46%)   Important fractures 1030 (16.4%)  Treating clinician:   - Emergency physicians: 3179 (50.6%) - Family physicians: 2173 (34.6%) - House staff: 936 (14.0%)   **Intervention period** N=6489   - Median age (range): 32 (18-100) - Female: 3105 (47.9%)   Important fractures 1082 (16.7%)  Treating clinician   - Emergency physicians: 3626 (55.9%) - Family physicians: 2031 (31.3%) - House staff: 832 (12.8%)   **Clinicians**  Not reported | Ottawa ankle rules |
|  | Stiell (2010)  **Country:** Canada  **Study design:** Randomised controlled trial | - Emergency physicians | Clinical practice guidelines + educational materials + educational meetings + reminders vs no intervention | 4531 patients: Adults presenting with a minor head injury who were alert and stable | **Patients**  **Intervention group**  **Pre-intervention period N=1049**   - Mean age (SD): 37 (19) - Female: 312 (29.7%)   **Mechanism of injury**   - Motor vehicle collision: 328 (31.3%) - Fall: 303 (28.9%) - Assault: 165 (15.7%) - Bicycle: 47 (4.5%) - Struck while pedestrian:34 (3.2%) - Struck in head: 77 (7.3%) - Sports: 83 (7.9%) - Other: 12 (1.1%)   **Post-intervention period N=1531**   - Mean age (SD): 40 (19) - Female: 449 (29.3%)   **Mechanism of injury**   - Motor vehicle collision: 450 (29.4%) - Fall: 459 (30%) - Assault: 252 (16.5%) - Bicycle: 99 (6.5%) - Struck while pedestrian: 81 (5.3%) - Struck in head: 94 (6.1%) - Sports: 81 (5.3%) - Other: 15 (1%)   **Control group**  **Pre-intervention period N=876**   - Mean age (SD): 39 (19) - Female: 251 (28.7%)   **Mechanism of injury**   - Motor vehicle collision: 251 (28.7%) - Fall: 240 (27.4%) - Assault: 158 (18%) - Bicycle: 62 (7.1%) - Struck while pedestrian: 54 (6.2%) - Struck in head: 44 (5%) - Sports: 63 (7.2%) - Other: 4 (0.5%)   **Post-intervention period N=1075**   - Mean age (SD): 41 (20) - Female: 317 (29.5)   **Mechanism of injury**   - Motor vehicle collision: 252 (23.4%) - Fall: 343 (31.9%) - Assault: 205 (19.1%) - Bicycle: 81 (7.5%) - Struck while pedestrian: 61 (5.7%) - Struck in head: 51 (4.7%) - Sports: 76 (7.1%) - Other: 6 (0.6%)   **Clinicians**  Not reported | Canadian CT Head Rule |
|  | Tajmir (2017)  **Country:** USA  **Study design:** Randomised controlled trial | - Medical doctors - Physician assistants | Reminders vs no intervention | 66 clinicians  613 patients (632 patient visits): Adults presenting with an ankle or foot injury | **Patient visits**  Intervention group N=258   - Seen by medical doctors: 162 (63%) - See by physician assistants: 96 (37%)   Control group N=374   - Seen by medical doctors: 265 (71%) - See by physician assistants: 109 (29%)   **Clinicians**  **Initial randomisation**  Intervention group N=32   - Medical doctors: 23 (72%) - Physician assistants: 9 (28%)   Control group N=34   - Medical doctors: 24 (71%) - Physician assistants: 10 (29%)   **Clinicians who saw patients**  Intervention group N=10   - Medical doctors: 4 (40%) - Physician assistants: 6 (60%)   Control group N=16   - Medical doctors: 12 (75%) - Physician assistants: 4 (25%)   **Mean years of experience**  Intervention group N=10   - Medical doctors: 19 years - Physician assistants: 10 years   Control group N=16   - Medical doctors: 18.5 years   Physician assistants: 9.3 years | Ottawa ankle rules |
|  | Wigder (1999) **Country:** USA  **Study design:** Single-arm trial | - Attending physicians - Junior doctors - Emergency nurse practitioners | Educational meetings + audit and feedback + educational materials + reminders vs no intervention | 27 physicians  362 patients: Adults presenting with a knee injury, defined as a contusion, fracture, sprain, ligament disruption, or traumatic effusion. | **Patients**  Pre-intervention period N=171  Intervention period N=191  **Clinicians**  Not reported | Ottawa knee rules |
|  | Zakhari (2016)  **Country:** USA  **Study design:** Single-arm trial | - Emergency nurse practitioners - Physician assistants - Attending physicians - Junior doctors | Interactive educational meetings + reminders vs no intervention | 100 clinicians | **Clinicians**   - Nurse practitioners: 25 (25%) - Physician assistants: 7 (7%) - Attending physicians: 11 (11%) - Postgraduate years 1: 1 (1%) - Postgraduate years 2: 5 (5%) - Postgraduate years 3: 2 (2%)   Registered nurses: 49 (49%) | Canadian CT Head Rule |

CT: Computed Tomography; ED: Emergency Department; IQR: Inter-Quartile Range; N: number of participants; N/A: Not Applicable; SD: Standard Deviation; MVA: Motor Vehicle Accident; NEXUS: National Emergency X-Radiography Utilization Study; UK: United Kingdom; USA: United States of America

**Supplementary Table 3. Risk of bias for each study as per the Cochrane EPOC risk-of-bias tool**

|  | Article | Random sequence generation | Allocation concealment | Baseline outcome measurements similar | Baseline characteristics similar | Incomplete outcome data | Knowledge of the allocated interventions adequately prevented during the study | Protection against contamination | Selective outcome reporting | Other risks of bias |
| --- | --- | --- | --- | --- | --- | --- | --- | --- | --- | --- |
|  | **Andruchow 2021** | Low risk | Low risk | Low risk | Low risk | Low risk | Low risk | High risk | Low risk | Low risk |
|  | **Ashurst 2014** | High risk | High risk | Low risk | Unclear risk | Unclear risk | High risk | High risk | Low risk | High risk |
|  | **Atkinson 2004** | High risk | High risk | Low risk | Unclear risk | Low risk | High risk | High risk | Low risk | High risk |
|  | **Auleley 1997** | Unclear risk | Low risk | Low risk | Low risk | Low risk | Low risk | Low risk | Low risk | Low risk |
|  | **Baker 2020** | High risk | High risk | High risk | High risk | Low risk | Low risk | High risk | Low risk | High risk |
|  | **Bessen 2009** | High risk | High risk | Unclear risk | Unclear risk | Low risk | High risk | High risk | Low risk | Low risk |
|  | **Cameron 1999** | High risk | High risk | Low risk | Unclear risk | Unclear risk | High risk | Low risk | Low risk | Low risk |
|  | **Gravel 2010** | Low risk | Low risk | Low risk | Low risk | Low risk | Low risk | Low risk | High risk | Low risk |
|  | **Griffith 2014** | High risk | High risk | Unclear risk | Unclear risk | High risk | Low risk | High risk | High risk | High risk |
|  | **Gwilym 2003** | High risk | High risk | Unclear risk | High risk | Low risk | High risk | High risk | Low risk | Low risk |
|  | **Helms 2023** | High risk | High risk | High risk | High risk | Low risk | High risk | Unclear risk | High risk | High risk |
|  | **Holroyd 2004** | Unclear risk | Low risk | Low risk | Low risk | Low risk | High risk | Low risk | Low risk | Low risk |
|  | **Kerr 2005** | High risk | High risk | Low risk | Low risk | Low risk | High risk | High risk | Low risk | Low risk |
|  | **Kim 2021** | High risk | High risk | High risk | High risk | Low risk | High risk | High risk | Low risk | High risk |
|  | **O'Sullivan 2006** | High risk | High risk | Unclear risk | High risk | Low risk | High risk | High risk | Low risk | High risk |
|  | **Rostas 2015** | High risk | High risk | Low risk | Low risk | Unclear risk | High risk | High risk | High risk | High risk |
|  | **Silveira 2016** | High risk | High risk | Low risk | Unclear risk | Unclear risk | High risk | Unclear risk | Low risk | Low risk |
|  | **Stiell 1995** | High risk | High risk | Low risk | Low risk | High risk | High risk | High risk | Low risk | High risk |
|  | **Stiell 2010** | Low risk | Low risk | Low risk | Low risk | Unclear risk | Unclear risk | Low risk | Low risk | Low risk |
|  | **Tajmir 2017** | Low risk | Low risk | Unclear risk | High risk | Unclear risk | High risk | Unclear risk | Low risk | High risk |
|  | **Wigder 1999** | High risk | High risk | Low risk | High risk | Unclear risk | High risk | High risk | Low risk | High risk |
|  | **Zakhari 2016** | High risk | High risk | High risk | High risk | Unclear risk | Unclear risk | Unclear risk | Low risk | High risk |
